# Between the Wars, Facing a Scientific Crisis: The Theoretical and Methodological Bottleneck of Interwar Biology: Introduction to Special Issue: New Styles of Thought and Practices: Biology in the Interwar Period

**DOI:** 10.1007/s10739-022-09689-2

**Published:** 2022-08-19

**Authors:** Jan Baedke, Christina Brandt

**Affiliations:** 1 Ruhr University Bochum, Bochum, Germany; 2 Friedrich Schiller University Jena, Jena, Germany

In 1919, the German zoologist Julius Schaxel began his book *Grundzüge der Theoriebildung in der Biologie* (Foundations of Theory Construction in Biology) with the following statement:

Physics and chemistry have maintained method and theory with increasing factual enrichment …. At the same time, biology loses its own reasoning, wants to be physics and chemistry of the living, collects immeasurable individual knowledge [Einzelwissen], hypotheses proliferate uncontrolled, self-reflection, methodology remains undone. Contemporary biology is in a state of crisis. A general biology, a science of life as such, exists in name only. (Schaxel 1919, p. 2; translation by the authors)

These worries about increasing specialization and the fragmentation of biology were widely shared by Schaxel’s colleagues in the early twentieth century (Ringer 1969; Harwood 1993; Nyhart 1995).^1^ During the interwar period – the short temporal bottleneck of twenty years (1918–1939) – debates about a crisis in biology were mainly fueled by two developments. First, new methodological advances and experimental techniques since the late nineteenth century (for example, the ability to separate developing blastomeres; microsurgical embryonic transplantation experiments; new experimental setups to study cytoplasmic inheritance [“*Dauermodifikation*”] and the norm of reaction of organisms; as well as novel methodologies to map chromosomes, identify and locate mutations on them, and trace genetic inheritance patterns) led to various new insights into the genetic and cellular nature of organisms. These approaches revealed organisms’ plasticity, robustness, regeneration, morphology, and inheritance (see, for example, Driesch 1892; Woltereck 1909; Morgan 1910; Sturtevant 1913; Stockard 1921; Griffith 1928; Spemann and Mangold 1924). However, interpreting this complex set of new data became increasingly difficult. For example, it was anything but transparent how developmental processes related to evolution or heredity, or which causal roles the newly introduced unit of the gene played in development or morphology. Second, after many decades of weighing the pros and cons of the most prominent theoretical positions of the time – vitalism and reductionism – theoretically-minded biologists felt that both dichotomous frameworks left a lot to be desired (see Allen 2005; Nicholson and Gawne 2015). These philosophical approaches provided little guidance for ordering new empirical findings and structuring theoretical and practical works.

Against this background, and at a time following the First World War that lacked a stable sociopolitical situation conducive to biological research, Schaxel and many of his cohorts, especially but not only in German-speaking countries, believed that biology needed a thorough reevaluation of its conceptual and theoretical foundations. This debate, moreover, should allow biology to structure and guide its different methodological approaches and interlink new empirical findings. They asked, among other things: What are the central concepts in biology, what is the subject of biological theory, and what is the appropriate experimental approach to scientifically understand life?

This special issue seeks to address the characteristics and changes in scientific thought and practices in this period of theoretical, empirical, and political crisis from historical and philosophical perspectives. This includes not only conceptual, methodological, and epistemological issues but also the question of how biological thought was situated geographically, institutionally, and politically during this time. So far, the historiography of the life sciences has been dominated by a strong focus on the historical developments in genetics and hereditary studies, molecular biology, eugenics, developmental biology, and evolutionary theories and related fields in order to understand main discourses in early to mid-twentieth century biology (see, for example, Olby [1974] 1994; Barkan 1992; Bowler 1992; Kohler 1994; Smocovitis 1996; Morange [1998] 2000; Kay 2000; Allen 2001; Laubichler and Maienschein 2007; Müller-Wille and Brandt 2016). However, as an increasing number of studies on the history of fields such as ecology, behavioral biology, marine biology, endocrinology, as well as plant breeding and agriculture suggest (see Elina et al. 2005; Wieland 2006; Logan 2007; Nickelsen 2009, 2014; Rumore 2012; Seyfarth and Zottoli 2015; Méthot 2016; Benson 2017; van de Grift and Foclaz 2017; Schürch 2017; Graf von Hardenberg 2019; Hill-Andrews 2019; Jax 2020), the first decades of the twentieth century saw a much wider spectrum of empirical approaches and theoretical debates. As one central development, attempts were made to found biology on the unit of the organism, which triggered a number of conceptual debates in early theoretical biology (among scholars like John Scott Haldane, Joseph Needham, Ludwig von Bertalanffy, Adolf-Meyer Abich, and Julius Schaxel). Besides this trend in biological theorizing, previous historiographies of the interwar period have so far often neglected a number of influential methodological research practices and institutional developments in fields outside of genetic and evolutionary trends, for example, in photosynthesis research and animal psychology.

Along these lines, this special issue seeks to offer a number of investigations – from “big picture” approaches to specific case studies – that pay tribute to the diversity of biological and philosophical views as well as their national, sociopolitical, and institutional contexts during the interwar period. In addition, several contributions discuss the relevance of interwar biology for today’s trends and challenges in the life sciences. Finally, this special issue has two focal points—theoretical biology and the organism, and experimental research practices towards reductionism—that mediate between the prevailing dichotomies of holism versus reductionism and organism versus (genetic) mechanism.

## Theoretical Biology and the Organism

After the clear historical break of the First World War, and driven not least by an empirically and conceptually opaque situation – a *Krise* as Schaxel and others labeled it – we find a period in which new attempts of scientists and philosophers to build theoretical biology increasingly flourished. This especially holds true for theoretically minded biologists in the United Kingdom and German-speaking contexts (Laubichler 2001; Amidon 2008; Nicholson and Gawne 2014, 2015; Peterson 2016; Müller 2017). A central idea for these scholars was to interlink developmental biology and embryology with evolutionary biology and (to some degree) with the emerging field of genetics by creating a unified conceptual framework that highlights the organism as the central methodological and explanatory starting point (Haraway [1976] 2004; Esposito 2015, 2017; Baedke 2019a; Jax 2020; Fábregas-Tejeda et al. 2021). This organicist movement (which included different holistic and dialectical approaches) represented a break in the dichotomous opposition between mechanism and vitalism (see Allen 2005), and, by integrating elements from both positions, was conceived as an alternative that could settle this fierce, long-lasting debate in the history of biology (Beyler 1996, p. 252; Haraway [1976] 2004, p. 2).^2^

While historians have recently begun to shed more light on these early days of theoretical biology, how many of the discussions emerged and developed is far from understood. This special issue asks which basic views—such as vitalism and reductionism, organicism, holism, and dialectical materialism—worked in the background of attempts to strengthen the conceptual framework of biology during this temporal bottleneck. The articles tackle many questions. What were the cross-national differences and similarities in these attempts? How did the chosen theoretical framework affect how the organism, especially its plasticity, was investigated? Was the organicist movement successful in replacing the vitalism-mechanism dichotomy? How does the trend towards theoretical biology resemble recent calls to renew the conceptual foundations of biology? And what can contemporary philosophy of biology learn from these past biotheoretical debates?

## Experimental Research Practices Towards Reductionism

Specific to the interwar period is not only a new wave of interest in a new theoretical or organismic biology but also new reductionist research programs. Whereas debates in philosophy and theoretical biology centered on concepts such as the “organism” or “life” in general, reductionist research, often accompanied by new interests in mechanisms, flourished in the 1920s and 1930s. Concerning such research in physical, chemical, and biological fields, historical investigations of the early twentieth century have often focused substantially on the rise of *Drosophila* genetics and early biochemical or biophysical developments leading toward molecular genetics. Research at the intersection of biology, chemistry, and physics in the 1920s and 1930s was often historically narrowed to form a kind of prehistory of 1940s and 1950s molecular biology. However, research during this time was way more diverse than that.

As the articles in this issue indicate, there were various new conceptual frameworks, technologies, and research practices in many often overlooked fields located at the physicochemical interface. Physiology, for example, was still an “umbrella field” for a variety of very different approaches, ranging from reductionist to even holistic research styles. In particular, mechanistic research approaches in the interwar period enabled new alliances and cross-disciplinary collaborations of chemists, biologists, and physicists in fields such as cell or plant physiology (Schürch 2017). Moreover, research interests in addressing basic phenomena of life from a mechanistic perspective went far beyond the historically well-known examples. Besides heredity and evolution, other fundamental properties of life such as metabolism, cellular respiration, and photosynthesis became highly dynamic research subjects. Research in these fields advanced new styles of transdisciplinary and molecular attempts in the interwar period (see, for example, Holmes 1991; Nickelsen 2015). How were these new physicochemical research communities formed and institutionalized? And how were experimental methodologies transmitted transnationally? These cases and questions convey a more diverse picture of early twentieth-century developments of biochemistry and biophysics, or the historical process of the “molecularization of biology,” more generally.

Moreover, a central question also concerns the relationship between experimental research dynamics and “styles of scientific thought” (Harwood 1993) in the 1920s and 1930s. How were scientific practices related to different views of the organism and other concepts? How did experimental practices contribute to a specific way of reasoning about life and the organism that became framed as *holistic*, *vitalist*, or *reductionist*? Again, the broad field of “physiology” at the time is an excellent example of how a variety of different new methods (quantitative or qualitative), research approaches (ranging from physicochemical to psychological and behavioral), and new concepts of the living being became intermingled. Debates in that field were much more complex than following a simple dichotomy of reductionism versus holism or organism versus mechanism. As we know from the classical studies of Harwood (1993), Sapp (1987), and others, the same is also true for the highly diverse fields of experimental studies on heredity and development in the interwar period, which were not yet dominated by a discourse on gene-centrism or reductionist experimental approaches in the interwar period.

These two focal points of the special issue on theoretical discussions of organism-centered views and their relation to research practices should lead to better understanding the diversity of biological and biophilosophical views in the early twentieth-century life sciences among different scientific communities and countries. These perspectives emerged from a workshop titled “New Styles of Thought and Practices in Early 20th Century Biology: Epistemologies and Politics” at Ruhr University Bochum (5–6 December 2016). Both the workshop and this special issue began with the idea to review and update Jonathan Harwood’s seminal book, *Styles of Scientific Thought: The German Genetics Community, 1900–1933*, nearly thirty years after its publication. We aimed to *build on* Harwood (1993) by keeping his fruitful focus on theoretical frameworks and new practices that emerged at the interface of reductionist and holist methodologies and against the background of conceptual debates between vitalism, mechanism, or organicism. Like Harwood, we focus largely on Central European life sciences (and German-speaking discourses) but seek to relate them by contrasting different research styles and theoretical debates in other national contexts. These transnational perspectives bridge Europe and the US (see the contributions by Julia Gruevska, Kärin Nickelsen, and Erik L. Peterson and Crystal Hall) and Germany and the UK (Jan Baedke).

At the same time, we seek to *update* Harwood’s analysis, which primarily focused on genetics and the German scientific context, by drawing on other disciplinary backgrounds and transnational developments in the interwar period. Our aim is to widen the understanding of interwar biology by looking at some underexplored debates and at disciplines apart from genetics. We explore the research questions, phenomena, theories, and methodological practices in various fields, such as animal psychology and neurophysiology (Gruevska), theoretical biology (Baedke, Peterson and Hall), developmental biology and experimental embryology (Christina Brandt’s two papers), as well as plant biology and physicochemical biology (Nickelsen). Several contributions highlight the transdisciplinary research movements in the interwar period, which interlinked biology with chemistry and physics and with philosophy and psychology. In addition, some of the contributors themselves take an interdisciplinary perspective at the intersection of history and philosophy.

The period covered by the five case studies in this issue is the twenty-year interwar interval, but they also consider relevant earlier and later developments. The methods employed by the authors range from archival research and conceptual analysis to tools from digital humanities. The first set of papers focuses on theoretical debates about organismic frameworks, vitalism, and holism. The paper by Erik L. Peterson and Crystal Hall, by applying topic modeling algorithms to over 30,000 journal articles, takes a longue durée perspective on the vitalism-mechanism debate. Following the thoughts of Joseph Needham and Francis Crick, they study whether this debate and the ideas defended by vitalism actually ended in the 1930s, as commonly believed. They argue that vitalism, in fact, never permanently dissolved and that even in times when there were no prominent and vocal champions of vitalism, discursive landscapes recurred that continued defending central vitalistic tenets.

Jan Baedke’s (2019a) paper addresses conceptual challenges that theoretical biologists had to face in the interwar period who tried to find a third way between vitalism and mechanism – a position that focused on the unit of the organism.^3^ By tracing central theoretical strands in this new organism-centered biology (that is, in British organicism, dialectical materialism, and [German] holistic biology), Baedke argues that theoretical biologists struggled with describing the individual organism as a causally autonomous and discrete unit and, at the same time, as inextricably interwoven with its environment. The paper then discusses theoretical biologists’ attempts to solve this tension between individualistic and anti-individualistic perspectives and argues that a similar conflict currently reemerges in evolutionary biology, as the field again tries to highlight the organism as a theoretical and explanatory starting point.

Christina Brandt’s two papers offer new historical perspectives on the work of the zoologist Hans Spemann against the scientific and political background of the time. The first paper focuses on Spemann’s joint work on newt merogones with Fritz Baltzer, a Swiss zoologist, at the intersection between heredity and development. Brandt shows that Spemann did not ignore research developments in heredity studies despite his well-known holistic and sometimes vitalistic position and his anti-reductionist rejection of *Drosophila* genetics. Instead, he made significant contributions to the debate. Brandt’s second paper discusses Spemann’s holistic research style in the context of broader theoretical developments in German discussions in the 1920s. She argues that contemporaries regarded Spemann as a representative of a “modern” way of doing biology precisely because of his anti-reductionist stance. Brandt also traces the metaphorical dynamics of the vague concept of an “organizer” within different research contexts and shows how the concept could unfold further vitalistic, materialist, and functionalist meanings in the 1920s and early 1930s.

With a similar focus on the interface between holistic and mechanistic research practices, Julia Gruevska’s paper looks closely at the transatlantic discourse between the Dutch animal psychologist and physiologist Frederik J. J. Buytendijk and the American neuropsychologist Karl S. Lashley in the late 1920s and early 1930s. This critical correspondence focused on the term *intelligence* as well as on the reliability of quantitative and qualitative methods. Gruevska uses this discussion as an exemplar to understand how the different holistic (phenomenological or hermeneutical) and reductionist theoretical frameworks clashed in interwar neurophysiology and how they shaped other experimental practices in animal behavioral research.

Kärin Nickelsen’s paper provides a detailed look into mechanistic methodologies in photosynthesis research during the interwar period. It shows how scientists trained in physics and chemistry rather than plant physiology established influential physicochemical methods that allowed studying the biochemical pathway of photosynthesis and the biophysics of the light reaction of this process. Nickelsen argues that in this development, knowledge of concepts and techniques from microbiology and human and animal biochemistry were increasingly transferred to the study of plant metabolism. By examining important cases in the search for the photosynthesis mechanism–including their relevant academic protagonists, institutional contexts, and investigative pathways–she elucidates how the underlying mechanistic framework allowed scientists not only to contribute to the emergence of interdisciplinary physicochemical biology, but also to venture into new and uncharted territory.

Overall, what these studies show is that while interwar biology could be labeled as being in a state of crisis–a crisis resulting from clashes between theoretical systems and underlying philosophical frameworks, between seemingly dichotomous research methodologies, between different disciplines and institutions, and between opposing political and national settings of research–this very crisis worked as an engine for theoretical and methodological innovations. It was a period that showed utmost awareness for important theoretical and conceptual challenges in biology (for example, about the nature of life and fundamental units of biological theorizing and explanation) that remain highly relevant today. At the same time, theorists’ goal to establish greater theoretical and conceptual order in biology did not constrict the field within a rigid methodological corset. On the contrary, by echoing the various dynamics in the social and political contexts that surrounded them, scientists were eager to question and overcome established disciplinary boundaries and to found new and long-lasting academic niches, both nationally and internationally. We hope this special issue will encourage more scholars to study this dynamic period and its role as a “creative bottleneck” in the history of twentieth-century biology.

## Footnotes

1 On Schaxel and his view of a crisis in biology, see also Hopwood (1997).

2 On whether the vitalism-mechanism debate was ever fully settled, see Peterson and Hall, this issue.

3 Baedke’s article (2019a) was included in *JHB* volume 52, issue 2 by mistake and should be regarded as part of this special issue collection of articles; see Baedke (2019b).
